# Investigation and experience sharing of increased Vancomycin-Resistant Enterococcus (VRE) cases in adult intensive care units of hospitals in southern Taiwan

**DOI:** 10.1017/ash.2025.134

**Published:** 2025-09-03

**Authors:** Yuen-Ju Chen, Yu-Ting Tsai, Huan-Yi Wu, Yuan-Ting Hong, Hui-Ping Lin

**Affiliations:** 1 Infection Control Office; 2 Section of Infectious Disease; 3 Nursing Department Pingtung Veterans General Hospital Pingtung City, Taiwan

## Abstract

**Background:** The adult intensive care unit comprises a total of 18 beds. On October 18, 2023, a text notification alerted us to a patient whose wound culture tested positive for Enterococcus faecium (VRE). Following protocol, an Anus VRE screening was conducted on the adjacent bed, revealing three additional positive cases, suggesting a cluster outbreak. Investigation and management were initiated. **Methods:** Through interviews, observations, medical record reviews, and expanded VRE screenings, a total of 8 beds tested positive, resulting in a positivity rate of 44.4% (8/18), all cases being colonization. Root cause analysis identified failures in hand hygiene among healthcare workers (HCWs), failure to wash hands before donning gloves, incorrect sequencing of environmental cleaning and disinfection, and inadequate implementation of contact isolation precautions. Measures included conducting Anus VRE screening for ICU admissions from October 15th to 18th, increasing the frequency of unit cleaning and disinfection, providing education and training, auditing hand hygiene practices and isolation measures, and centralizing VRE patient care. **Results:** Utilization of multiple measures for controlling drug-resistant bacterial infections, including auditing hand hygiene, environmental cleaning and disinfection, implementing contact isolation precautions, and conducting environmental sampling, yielded negative results. Observation until November 30th showed no new cases, effectively controlling the spread of drug-resistant bacteria and preventing healthcare-associated infections due to VRE. **Discussion:** Despite HCWs’ often busy clinical care responsibilities leading to neglect of hand hygiene or substituting handwashing with glove usage, and lapses in implementing contact isolation precautions, no healthcare-associated infections occurred, and patients were successfully discharged without disease exacerbation or fatalities. Environmental sampling was conducted post-environmental disinfection. Additionally, all VRE-positive patients were identified as Enterococcus faecium (VRE). Due to limitations, PFGE testing couldn’t be conducted, hence strain and susceptibility determination confirmed the same VRE colonization event within the hospital.

